# Impaired Function of the Blood-Testis Barrier during Aging Is Preceded by a Decline in Cell Adhesion Proteins and GTPases

**DOI:** 10.1371/journal.pone.0084354

**Published:** 2013-12-31

**Authors:** Catriona Paul, Bernard Robaire

**Affiliations:** 1 Department of Pharmacology and Therapeutics, McGill University, Montréal, Canada; 2 Department of Obstetrics and Gynecology, McGill University, Montréal, Canada; Inserm, France

## Abstract

With increasing age comes many changes in the testis, including germ cell loss. Cell junctions in the testis tether both seminiferous epithelial and germ cells together and assist in the formation of the blood-testis barrier (BTB), which limits transport of biomolecules, ions and electrolytes from the basal to the adluminal compartment and protects post-meiotic germ cells. We hypothesize that as male rats age the proteins involved in forming the junctions decrease and that this alters the ability of the BTB to protect the germ cells. Pachytene spermatocytes were isolated from Brown Norway rat testes at 4 (young) and 18 (aged) months of age using STA-PUT velocity sedimentation technique. RNA was extracted and gene expression was assessed using Affymetrix rat 230 2.0 whole rat genome microarrays. Microarray data were confirmed by q-RT-PCR and protein expression by Western blotting. Of the genes that were significantly decreased by at least 1.5 fold, 70 were involved in cell adhesion; of these, at least 20 are known to be specifically involved in junction dynamics within the seminiferous epithelium. The mRNA and protein levels of *Jam2*, *Ocln*, *cdh2* (N-cadherin), *ctnna* (α-catenin), and *cldn11* (involved in adherens junctions), among others, were decreased by approximately 50% in aged spermatocytes. In addition, the GTPases *Rac1* and *cdc42*, involved in the recruitment of cadherins to the adherens junctions, were similarly decreased. It is therefore not surprising that with lower expression of these proteins that the BTB becomes diminished with age. We saw, using a FITC tracer, a gradual collapse of the BTB between 18 and 24 months. This provides the opportunity for harmful substances and immune cells to cross the BTB and cause the disruption of spermatogenesis that is observed with increasing age.

## Introduction

Increasing age in men is characterized by a decrease in spermatozoal motility, normal morphology and a decline in the production of functional spermatozoa that produce healthy offspring [1]–[4]. The aging rat testis is characterized by the loss of germ cells, the appearance of Sertoli cell only tubules and thus testicular atrophy or regression [5]. However, the reason for testicular regression during aging remains largely unknown though there are a number of possibilities that include changes in the blood-testis barrier (BTB). This barrier, despite its name, is a protective barrier only for the post-meiotic germ cells within the seminiferous epithelium and not to the entire testis. Maintenance of normal seminiferous epithelium function and morphology is facilitated by dynamic associations or junctions between the germ cells and Sertoli cells (reviewed in [6]). These cell adhesions not only tether the cells of the seminiferous epithelium together but also assist in the movement of germ cells across the BTB, which is situated between the pre-leptotene and pachytene spermatocytes; it reduces exposure of the post-meiotic germ cells to harmful chemicals that may be present in the systemic circulation and protects the spermatids, which express ‘foreign’ proteins, from immunological attack (reviewed in Mital et al [7]). There are a number of different types of junctions present in the seminiferous epithelium including tight junctions, gap junctions and adherens junctions; all of which function collectively in maintaining the BTB. This study focuses on the basal epithelial junctions: adherens junctions (including ectoplasmic specializations) and tight junctions.

As one of the main morphological features of the aging testis is regression due to germ cell loss, the question as to what is responsible for causing this loss is central. Some of the theories proposed to account for this effect include: an accumulation of DNA damage in the spermatogonial stem cells (SSCs), an age-associated decrease in the support of the Sertoli cells and thus the nurturing niche of the SSCs is no longer able to support the maintenance and division of these cells, a breakdown of the BTB, or some combination of all three. We have previously shown that, by 24 months of age in the Brown Norway rat, the BTB has become compromised [8]. Lanthium tracer penetrated into the lumen of the seminiferous tubule in 24 month old rats but not in 3 month old rats. The seminiferous epithelium still has a full complement of germ cells in rats at 18 months of age; testicular atrophy does not occur until around 20 months. We propose that, with age, there is a gradual deterioration of the cell adhesions and junctions that preserve the integrity of the adlumninal compartment of the seminiferous tubules and that this is preceded by a decline in the junctional proteins themselves. To test this hypothesis we did microarray analysis on germ cells isolated from 18 month old rats that are at the onset of germ cell loss and tracer studies on rats from three different age groups to determine whether there are gradual changes in the BTB with age.

## Materials and Methods

### Animals

Male Brown Norway rats of 4, 18, 21 and 24 months of age (4–6 animals per group) were maintained under standard conditions as described in *A Guide to the Care and Use of Experimental Animals* prepared by the Canadian Council on Animal Care. All animals were kept on a 12L:12D cycle with free access to food and water. all procedures were approved by the Animal Care and Use Committee of McGill University (McGill Animal Resources Centre protocol #4687). Rats in the ‘young’ group (4 months old) were purchased directly from Harlan (Indianapolis, IN), while rats in the ‘aged’ groups were purchased from Harlan via the National Institute on Aging (Bethesda, MD). The ‘aged’ rats at 18 months were chosen as this is the time point just prior to the onset of germ cells loss and testicular atrophy and the 21 month old rats were chosen as at this time they are undergoing testicular regression. The 24 month old rats are mostly in a state of regression.

### Germ Cell Separation

Each rat (4 and 18 months) was examined for the presence of regressed testes (<1.4 g) by palpation followed by weighing after excision, and only those rats that did not have regressed testes were used for cell separation. Animals were euthanized by CO_2_ asphyxiation. Spermatogenic cells were obtained through cell separation using the STA-PUT velocity sedimentation technique as described by Bellve et al [9] and modified by Aguilar-Mahecha et al [10]. Briefly, the tunica albuginea was removed along with any large blood vessels; the parenchyma was subjected to enzymatic digestion at 34°C, first with 0.5 mg/ml collagenase (Sigma Aldrich Canada, Oakville, ON, Canada) for 16 min followed by sedimentation and washing then with 0.5 mg/ml trypsin (Type I; T8003; Sigma) and DNase I (Type I, DN-25; Sigma) for 16 min. After dissociation, cells were filtered through a nylon mesh (70 µm) and washed with RPMI (RPMI medium 160; Invitrogen, Burlington, ON, Canada) containing 0.5% BSA. Cells were centrifuged and filtered and 5.6×10^8^ cells in 25 ml of 0.5% BSA (bovine serum albumin)/RPMI were loaded into the velocity sedimentation apparatus (STA-PUT; Proscience, Don Mills, ON, Canada), and separated on a 2–4% BSA gradient in RPMI for separation by sedimentation at unit gravity. Fractions of pachytene spermatocytes were identified by phase contrast microscopy, pooled, aliquoted at 5×10^6^/ml, pelleted and stored at −80°C until use [11].

### RNA Extraction and Gene Expression Microarray Analysis

Total RNA was extracted from pachytene spermatocytes (∼5×10^6^ cells) using the RNeasy Mini Plus kit with on-column DNase digestion (Qiagen, Mississauga, ON, Canada). RNA concentrations were determined using the Nanodrop 2000 (Nanodrop Technologies, Wilmington, DE) and quality assessed using a Bioanalyzer (Agilent Technologies, Santa Clara, CA). Gene expression analysis was done using Affymetrix Rat 230 2.0 microarrays (n = 6 per group) in collaboration with Genome Quebec. Three µg of RNA was reverse transcribed and the cRNA was hybridized on the microarray according to the manufacturer’s instructions. All chips had a background signal of less than 75 and *Gapdh* 3′/5′ hybridization rate from 0.91 to 1.6. The Robust Multiarray Alogarithm (RMA) [12] was applied and only those genes with an expression value higher than 50 were considered as ‘expressed’. The raw data obtained were further normalized per gene to the median (Genespring v11.0, Agilent Technologies). All GC- RMA data were placed in GEO (Accession no. GSE29963, NCBI). Statistical significance between the two groups was tested by the student’s t-test using a *p* value of <0.05 and the Benjamini-Hochburg post-hoc test. Probe sets that were significantly altered were further filtered using a minimum 1.5-fold difference. Cellular pathways were obtained using Pathway Studio 9 (Elsevier).

### Real-time Quantitative Reverse Transcriptase-PCR (q RT-PCR)

RNA was diluted to a working concentration of 2 ng/µl and Quantitect One-Step SYBR Green quantitative RT-PCR (Qiagen) was done using the Roche Lightcycler (Roche Diagnostics, Laval, QC, Canada) according to the manufacturer’s instructions. PCR thermal cycling parameters were: 95°C for 15 min (one cycle), 94°C for 15 sec, 55°C for 30 sec, and 72°C for 30 sec (50 cycles). Standard curves were generated using 0.1, 1, 10, and 100 ng/ml of RNA from control pachytene spermatocytes in each run for quantification. RT-PCR primers (Table 1) were either ready-made Quantitect Primer Assays (Qiagen) or designed using Primer3 software (http://frodo.wi.mit.edu) and provided by Alpha DNA (Montreal, QC, Canada). The mRNA expression level of the FSH receptor was also determined to assess whether the isolated pachytene population was contaminated by Sertoli cells. The expression levels of all genes of interest were corrected using an endogenous control, 18S rRNA, and the fold difference in mRNA expression of the samples was determined. The results shown are the means of at least six rats per group, done on two separate occasions and each sample was analyzed in duplicate.

**Table 1:** Primers used in qRT-PCR.

Gene Name	Accession #	Quantitect primer ref #
Vcl	NM_001107248	QT00428925
Ctnna	NM_001106598	QT01292340
Jam2	NM_001034004	QT01302616
Ocln	NM_031329	QT00196357
Cldn11	NM_053457	QT00176148
Profilin	NM_030873	QT00181517
Ncad	NM_031333	QT00185262
RhoB	NM_022542	QT00366919
Rac1	NM_134366	QT01620003
Cdc42	NM_171994	QT00190288
Rab9	NM_053458	QT00191345
citron	NM_001029911	QT01079897
Chn2	NM_032084	QT00179046
Fshr	NM_199237	QT00459760
	**Forward**	**Reverse**
18S	CCTCCAATGGATCCTCGTTA	AAACGGCTACCACATCCAAG

### Immunohistochemistry

Rats were asphyxiated with CO_2_ and testes were excised, the ends were punctured with a 26 gauge needle and then immersed in modified Davidson’s fixative [13]. After 2 h of immersion fixation the testes were cut in half to allow better fixation and replaced in the fixative for a total of 24 h. The testes were then dehydrated in a series of alcohol solutions and embedded in paraffin. Testicular sections (5 µm) were cut and mounted on charged slides. Slides were dewaxed using xylene and rehydrated. Non-specific binding sites were blocked using normal goat serum (NGS; Vector Laboratories, ON, Canada) diluted 1∶4 in BSA/TBS (Tris Buffered Saline) (3%, w/v) for 30 min. Sections were incubated overnight at 4°C with the primary antibodies specific for VCL. JAM2, CDC42 and CTNNA at 1∶50 NGS/TBS/BSA; control sections were incubated with blocking serum alone. Following washing, sections were incubated with Alexa-546 or Alexa-488 conjugated goat anti-rabbit or goat anti-mouse secondary antibodies respectively, washed and counterstained with DAPI. Images were captured using a Leica microscope DM LB2 (Leica, ON, Canada) under a 40x objective and micrographs were captured with an Infinity-3 video camera (Lumenera, ON, Canada).

### Western Blotting

Total protein was extracted from pachytene spermatocytes using RIPA lysis buffer and concentration was determined using the BioRad protein assay kit according to manufacturer’s instructions (BioRad, Mississauga, ON, Canada). Samples (10 µg/lane) were resolved by SDS polyacrylamide (w/v) gradient (4–12%) gels (Invitrogen) at 150V for 1.5 h then transferred onto PVDF membranes. Membranes were blocked with 5% nonfat milk in PBS containing 0.1% Tween-20. Proteins were detected using antibodies specific for N-CAD (ab18203, 1∶500), CTNNA2 (ab76015, 1∶500), CHN2 (ab59625, 1∶500), RAB9 (ab2810, 1∶500), RAB10 (ab104859, 1∶500), CDC42 (ab64533, 1∶500), RHOB (ab68827, 1∶500), RAC1 (ab33186, 1∶500; all Abcam, Cambridge, MA), VCL (V9131, 1∶500; Sigma) and OCLN (711500, 1∶500; Invitrogen), all diluted in 3% nonfat milk in TBS-0.1% Tween followed by HRP-linked secondary antibodies and normalized against the amount of β-actin (sc1616, 1∶5000, Santa Cruz) to correct for loading.

### Assessment of the Integrity of the Blood-testis Barrier

A group of 4 month old rats received CdCl_2_ treatment (3 mg/kg bw, i.p.) which disrupts the BTB, to serve as a positive control [14] for the BTB integrity assessment. Three days later both treated and untreated rats at 4, 18, 21 and 24 months old (n = 3) were anesthetized and an incision (0.5 cm) made over the jugular vein to expose the vessel. Using a 26 gauge needle, an infusion of FITC-conjugated inulin (Sigma Aldrich) in a final volume of 200 µl PBS was administered to each rat via the jugular vein. The surgical area was then stitched with sterile silk braided black suture (Ethicon, Inc., Somerville, NJ, USA), and the rats were allowed to recover for 90 min before being killed by carbon dioxide asphyxiation. Both testes were immediately removed and frozen in OCT (Electron Microscopy Sciences, Hatfield, PA, USA) on dry ice. Frozen sections of ∼6 µm were cut in a cryostat and examined on a Leica (DM-LB2) microscope equipped with an Infinity 3 camera (Luminera, Ottawa, ON, Canada). FITC fluorescence in the seminiferous epithelium in each age group and in positive (CdCl_2_ treatment) controls was photographed using Infinity Capture software (Lumiera). Images were captured for at least 20 tubules from each rat and analysed using Image J software (NIH, Bethesda, MD). The distance that the fluorescence (FITC) had diffused from the basement membrane into each tubule for each age group was determined and was used as a measure of BTB integrity.

### Statistical Analysis

Results expressed as means and standard errors of the mean were analyzed by Student’s t-test in all cases with the exception of the tracer study data which was analyzed by one-way ANOVA. This was done using GraphPad Prism version 5 (Graph Pad Software Inc., San Diego, CA).

## Results

### Genes and Proteins Involved in Cell Adhesion are Altered in Aging Spermatocytes

**Figure 1:**
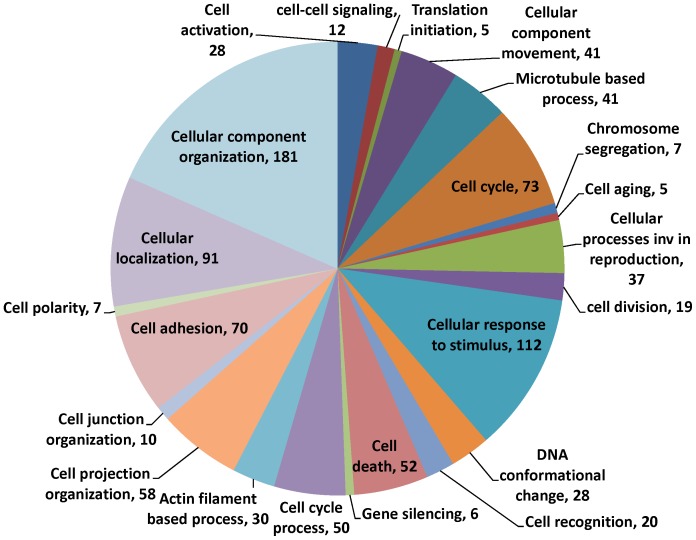
Gene ontology analysis. Analysis of genes that are at least 1.5-fold changed in aged pachytene spermatocytes compared to young. The pie chart shows the prevalence of different GO terms in the cellular processes category in relation to aging.

**Figure 2:**
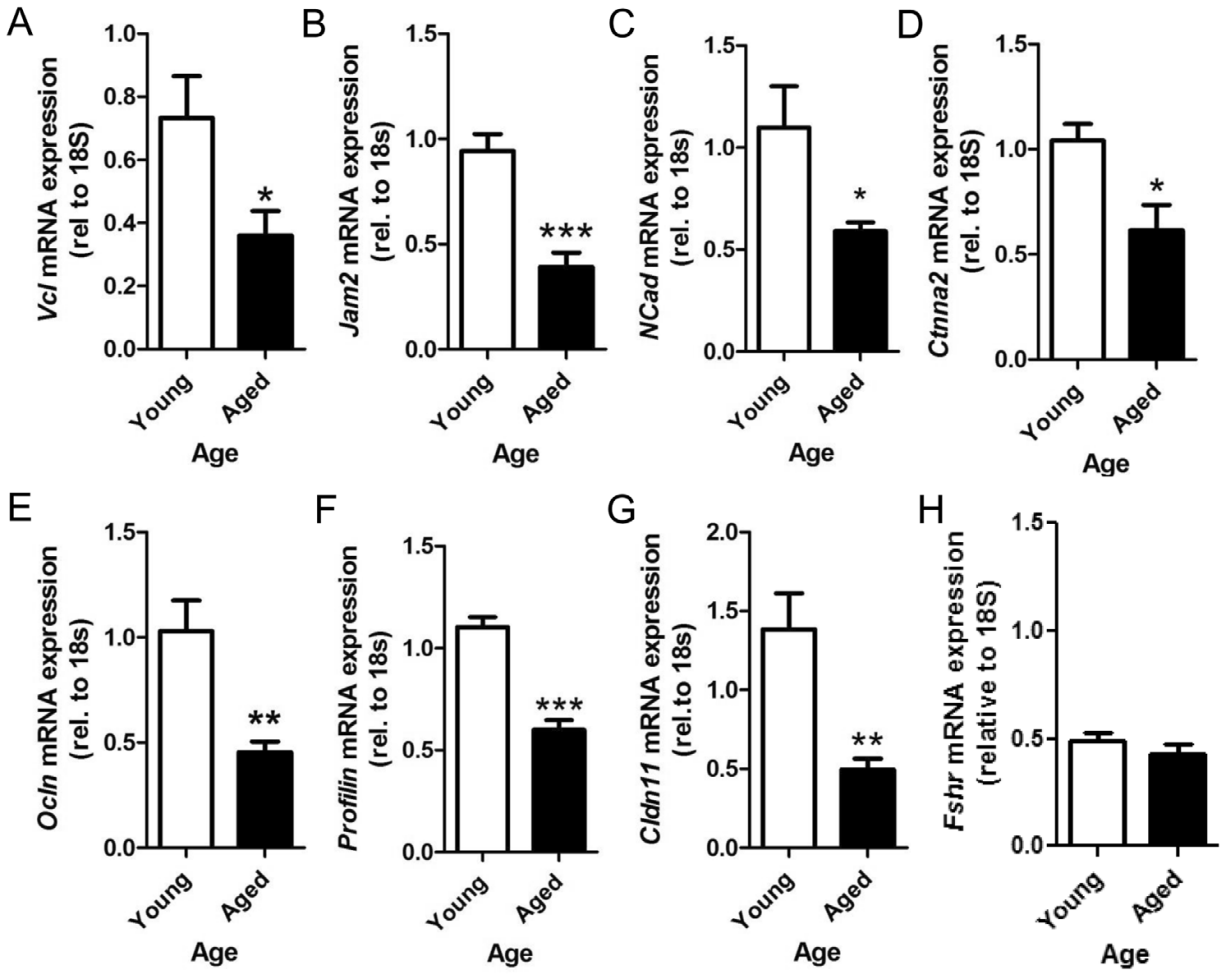
Relative mRNA expression of junction components. (A) Vcl, (B) Jam2, (C) *N-cad*, (D) *Ctnna*, (E) *Ocln* (E), (F) *Profilin*, and (G) *Cldn11* in young and aged pachytene spermatocytes. Relative mRNA expression of *Fshr* (H). *p<0.05, **P<0.01 and ***p<0.001. n = 5.

**Figure 3:**
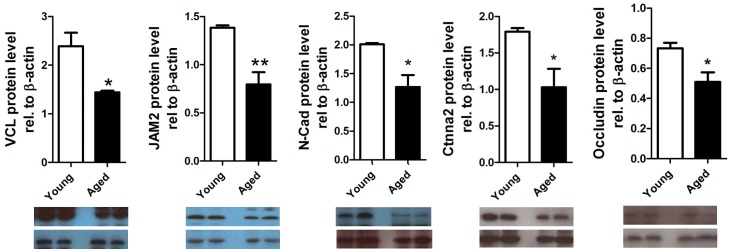
Relative expression of the junction proteins. (A) VCL, (B) JAM2, (C) N-Cad, (D) Ctnna and (E) Ocln in young and aged pachytene spermatocytes. *p<0.05, **P<0.01 and ***p<0.001. n = 5.

Whole rat genome Affymetrix 230 2.0 microarrays were used to assess the impact of age on gene expression in pachytene spermatocytes. Of the 31,099 probesets on the array, 522 were altered by at least 2-fold (463 down and 59 up) and more than 1000 were altered by at least 1.5-fold. GC- RMA data are available in GEO (Accession no. GSE29963, NCBI). Gene ontology (GO) analysis on the significantly altered transcripts, revealed that more than 20% of the transcripts in the ‘cellular processes’ category were related to cell adhesion, cell junctions, cell localization and cell polarity. More specifically, 70 transcripts were involved in cell adhesion, 68 in junction and projection organization, 30 in actin filament-based processes and 7 transcripts in cell polarity (Fig. 1). From the microarray results a selection of 7 transcripts associated with junctions in the testis were chosen to be confirmed by qRT-PCR. These transcripts were chosen to cover different types of junctions and functions in the BTB encompassing ectoplasmic specializations (adherens junctions), tight junctions and also the GTPases that are involved in these junctions (see summary Table 2). The transcripts chosen were Vcl, Ctnna2, Jam2, Ocln, Cldn11, Profilin and Ncad and all transcripts were significantly downregulated in the aged pachytene spermatocytes in comparison to the young (Fig. 2). The effect of aging on the expression of five of the corresponding proteins, VCL, CTNNA2, JAM2, OCLN and NCAD, was determined (Fig. 3); protein levels were also significantly decreased by 1.7-fold, 1.7-fold, 1.7-fold, 1.4-fold and 1.6-fold, respectively. There was thus an overall decrease in the expression of genes and proteins associated with junction dynamics in the aging spermatocytes. It is noted that although occludin is routinely found to be expressed in spermatocytes at the transcript level, it was not expected that occludin protein would also be present as this is historically known as a Sertoli cell protein. Therefore the protein detected in our samples may have come from the small amount of Sertoli cells present within the isolated germ cells. Although some degree of Sertoli cell contamination was confirmed by a low level of mRNA expression of the FSH receptor in our cells (Fig. 2H), the extent of that contamination was similar as a function of age.

**Table 2:** Summary of transcripts and/or proteins studied with the type of junction they are associated with.

**Adherens Junctions**	Vinculin (Vcl)
	α-Catenin (Ctnna2)
	N-cadherin (Ncad)
	Chimaerin (Chn) 2
	RhoB (GTPase)
	Rac1 (GTPase)
	Cdc42 (GTPase)
	Rab9 (GTPase)
	Rab10 (GTPase)
	Citron (Cit)
**Tight Junctions**	Junction Adhesion Molecule (Jam) 2
	Occludin (Ocln)
	Claudin 11 (Cldn11)
**Actin cytoskeleton**	Profilin (Pfn)

### Aging causes Changes in GTPase Gene Expression and Protein Levels

**Figure 4:**
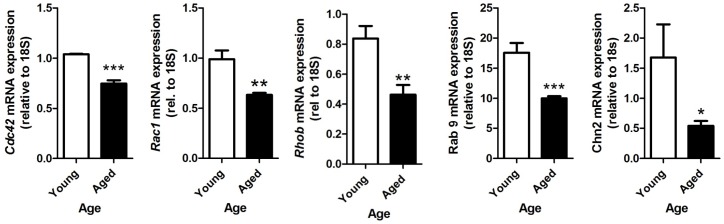
Relative mRNA expression of the GTPases in young and aged pachytene spermatocytes. (A) *Cdc42*, (B) *Rac1*, (C) *RhoB*, (D) Rab9, and (E) *Chn2*. *p<0.05, **P<0.01 and ***p<0.001. n = 5.

**Figure 5:**
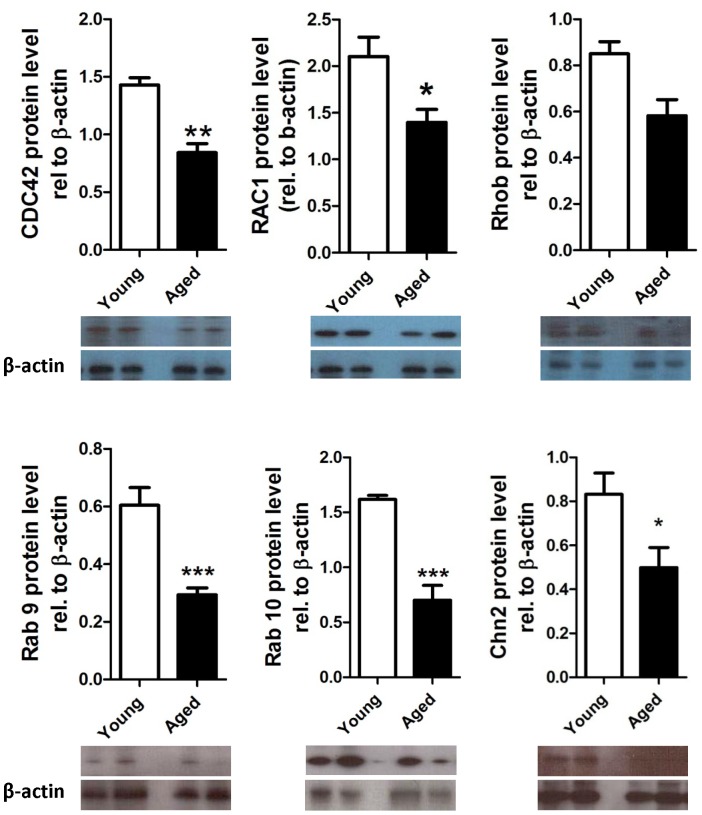
Relative protein expression of GTPases in young and aged pachytene spermatocytes. (a) CDC42, (B) RAC1, (C) RhoB, (D) Rab9, (E) Rab10, and (F) Chn2 (F). *p<0.05, **P<0.01 and ***p<0.001. n = 5.

We also noted that the expression of a number of genes for GTPases and GTPase regulators was altered in aged spermatocytes. GTPases and their effector proteins control assembly and recruitment of some of the junction proteins, and vice versa, in the testis [15], as reviewed in [16]). Microarray results were confirmed by qRT-PCR for Cdc42, Rac1, RhoB, Rab9 and Chn2; they were all significantly downregulated in the aged pachytene spermatocytes in comparison to the young (Fig. 4). CDC42, RAC1, RHOB, RAB9, RAB10 and CHN2 protein levels were also significantly decreased by 1.7-fold, 1.5-fold, 1.5-fold, 2.0-fold, 2.3-fold and 1.7-fold, respectively (Fig. 5). Therefore, in addition to the downregulation of junction-associated proteins, we also saw an overall decrease in the expression of some of the factors that are thought to control the formation and maintenance of these junctions, the GTPases.

### Large Networks of Cell-adhesion Related Proteins are Altered in Aging Spermatocytes

**Figure 6:**
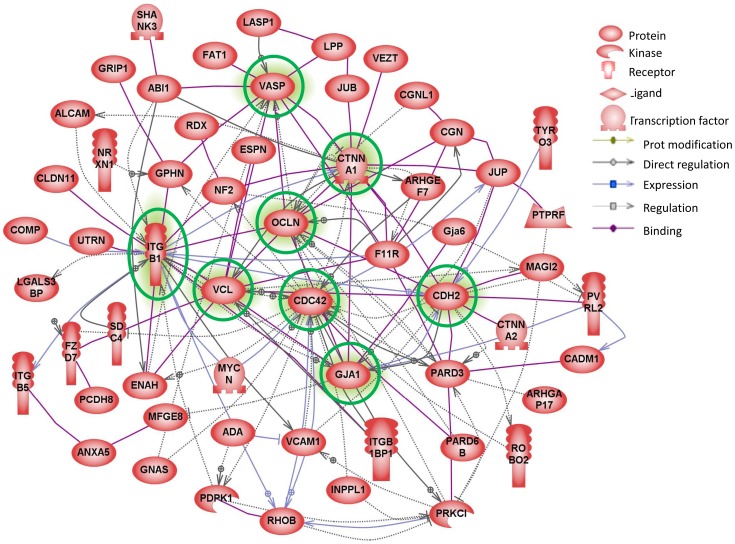
Gene linkage analysis. Direct linkages between genes that are identified by GO analysis to be involved in cell adhesion and junction dynamics and significantly changed in the aged spermatocytes compared with the young. Genes highlighted in green are those with the highest number of interactions and connectors.

Out of the 120 transcripts identified in the microarray to be involved in cell adhesion, cell polarity and junction dynamics, 58 were recognised by pathway analysis to have direct interactions (Fig. 6). Proteins highlighted in green are those with the highest number of interactions and connectors and are thus thought to be integral in the changing architecture of cell adhesions within the aging testis. The proteins with the highest number of connections, i.e. more than 10 interactions each, and that seemed central to those that change with age were CDC42, CTNNA1, OCLN, VCL, ITGβ1, GJA1, CDH2 and VASP. CDC42, a small GTPase, and IGβ1, an important junction protein, both of which are involved in adherens junctions, were the two ‘most connected’ genes/proteins, having 24 and 22 interactions, respectively (Fig. 6).

### Aging causes Changes in the Expression of BTB Proteins

**Figure 7:**
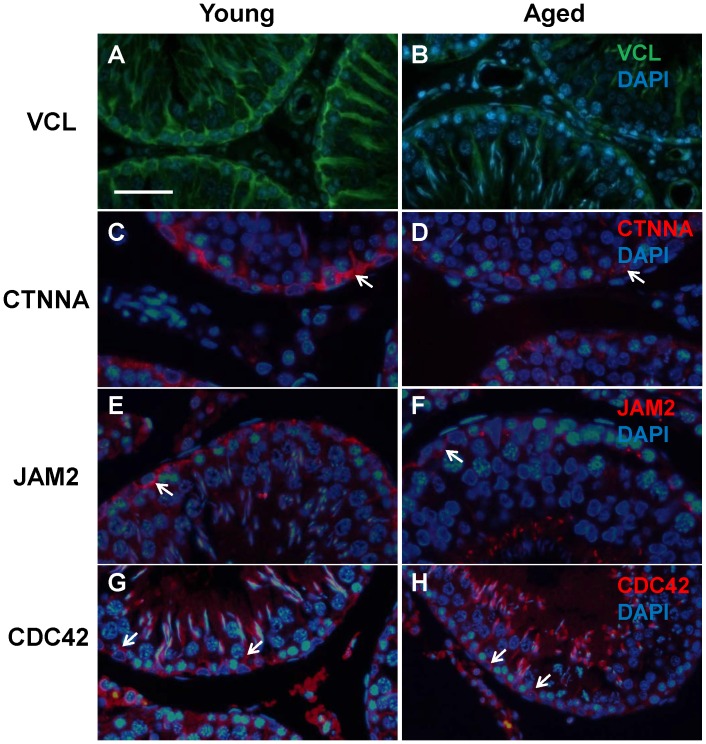
Changes in immunoreactivity of junction-associated proteins that were altered in the microarray during aging. Localization of VCL (A, B), CTNNA (white arrows; C, D), JAM2 (white arrows; E, F) and CDC42 (white arrows; G, H) in the seminiferous epithelium of young and aged rats (n = 4 per group) counterstained with DAPI. All four proteins were found to be localized to the BTB in the basal compartment of the seminiferous tubules at both ages with a weakening of expression in the aged testes. Bar = 50 µm.

Vinculin (VCL) is involved in the ectoplasmic specializations, a type of adherens junction, in the adult rat testis [17]. A strong continuous expression of VCL was observed around the basal cells at the location of the BTB in the young testes and also between Sertoli cells as projections towards the lumen (Fig. 7A). However, in the aged testis this expression not only seemed diminished but also did not appear to be continuous at the barrier, with more of an intermittent staining despite having the same localization (Fig. 7B). Alpha catenin (CTNNA) is also involved in adherens junctions and may play a role in linking tight junction and adherens junction proteins [18]. In the testes from young animals, CTNNA2 was localized around the cells at the basement membrane of the seminiferous tubule at the location of the BTB (Fig. 7C). Again, in the aged testes, the immunofluorescence was weaker and exhibited a more intermittent staining rather than a continuous expression (Fig. 7D). Jam2, a tight junction protein, was seen to have a similar localization as Ctnna2 at the location of the BTB in the young testes (Fig. 7E) and a lower intensity in the aged (Fig. 7F). Cdc42 is a small GTPase that is involved in BTB integrity and was found to be prominently expressed near the basal membrane at the site of the BTB in young testes (Fig. 7G). In aged testes, Cdc42 immunoexpression around the BTB was less detectable although still present (arrows, Fig. 7H).

### The Impact of Age on the BTB

**Figure 8:**
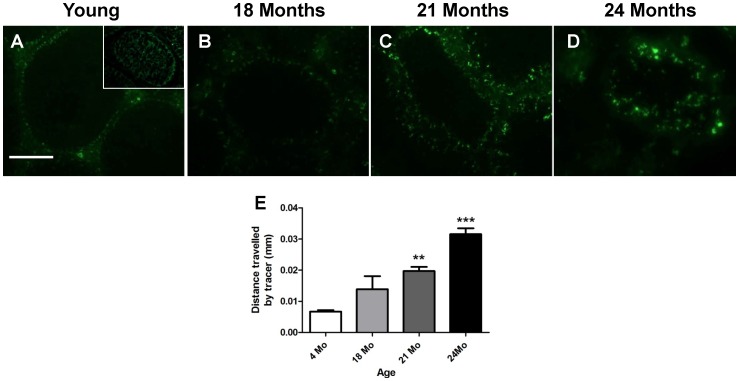
An in vivo functional assay to determine the integrity of the blood-testis barrier (BTB) during the course of aging. Localization of inulin-FITC (green fluorescence) in frozen, OCT-embedded sections from young (4 months) and aged (18, 21 and 24 months) rats. The functionality of the BTB is determined by the distance travelled within the seminiferous tubules as a functional BTB would block diffusion of FITC tracer from the basal to the luminal compartment as seen in young testes (A, B). When the BTB is not fully functional the tracer passes further toward the lumen of the seminiferous tubule, as seen in the aged testes (C–D) and in the CdCl_2_-treated testes (inset). The distance travelled, by the tracer, from the basement membrane was measured for each age group (E). n = 3, **P<0.01, ***P<0.001. Bar = 100 µm.

In order to determine if, with age, the efficiency of the BTB deteriorates, an *in vivo* functional BTB assay was used. A tracer with a fluorescent tag (FITC) was administered via the jugular vein and the distance of tracer diffusion across the seminiferous epithelium was determined as a measure of the integrity of the BTB. In the positive control (CdCl_2_ -treated), the tracer passed through the BTB and reached the lumen of the seminiferous tubule (Fig. 8A inset) so that the entire tubule was FITC-positive. In the 4 month old rats, the FITC tracer was effectively blocked at the BTB and only diffused up to the first layer of cells (Fig. 8A and E), which is the approximate location of the BTB. By 18 months of age, diffusion of the tracer increased slightly and it travelled double the distance of that in the 4 month group, though this was not significant (Fig. 8B and E). However, at 21 and 24 months of age, the distance of tracer diffusion across the BTB was significantly increased, by 2.8 and 4.7 fold respectively, in comparison to the 4 month old rats (Fig. 8C–E). These data suggest that the BTB gradually loses efficiency from 18 months onwards.

## Discussion

In this study, we explored the possibility that the BTB that normally protects germ cells, is compromised during the aging process, and determined whether this allows the passage of harmful substances that would otherwise not be able to enter the seminiferous tubules past the pre-leptotene spermatocytes. The junction and cell adhesion proteins within the testis not only help in the maintenance of structural integrity but also facilitate many of the events that occur during spermatogenesis such as meiosis and spermiation (reviewed by Wong et al. [19]). Any changes that occur to these structures would undoubtedly result in a decline in the process of spermatogenesis. It was our aim here to determine whether there were changes in the components of the seminiferous tubular junctions with age.

In our study, many of the transcripts and proteins, that make up the BTB showed decreased levels in the aged spermatocytes. In particular, we saw that there was a decrease in many of the transcripts and proteins that are involved in adherens junctions, such as the ectoplasmic specializations, including VCL, ITGβ1 and N-cadherin [20]. This type of junction has long been considered to form interactions between Sertoli cells and pachytene spermatocytes [21]. The β integrins, and specifically β1-integrin, are essential for the dynamics of ectoplasmic specializations [22]; β1-integrin was considerably reduced in the aged spermatocytes compared to the young. There have not been many studies looking at the effects of aging on the junctions present within the testis though there have been a number done on other tissues. For example, in both spinal ganglia (Procacci, 2008) and osteoblasts [23] there is a decrease in the ability to form gap junctions with age. In addition to the decrease we saw in proteins involved in adherens junctions, a range of small GTPases that are involved in junction assembly, e.g. in the recruitment of cadherins to the adherens junctions found between Sertoli and germ cells, were similarly decreased in aged spermatocytes. It would be of interest to determine the changes within Sertoli cells with age; however few studies have been done on Sertoli cells isolated from adult rats [24], and we are aware of none using aged rats. The majority of previous work has been done on pre-pubertal rats, where it is straightforward to obtain a population of Sertoli cells, with little or no contaminated with germ cells, up to the time of the formation of the blood-testis barrier; however, attempts in our lab at isolating Sertoli cells from aged rats have not been successful due to increased cell fragility with advancing age.

GTPases are small proteins that shuttle between active (GTP-bound) and inactive (GDP-bound) states and that act as molecular switches controlling molecular events such as actin reorganization [25]. They also help in the recruitment of adhesion proteins [26], cell polarity and cell movement events such as movement of germ cells (reviewed in [27]). It has been proposed that the Sertoli-germ cell adherens junctions (e.g. ectoplasmic specializations), but not tight junctions, are regulated by the GTPase RHOB [28], a small GTPase from the Ras superfamily of Rho GTPases, which in our study was reduced in the aged spermatocytes to around 50% of that in the young spermatocytes. Two other such GTPases, CDC42 and RAC1, were also downregulated in the aged spermatocytes. Both Cdc42 and Rac1 are thought to regulate actin distribution [29] and recruitment of junction proteins (reviewed in [30]). RAP1, which regulates both CDC42 and RAC1 GTPases [31] [32]was also downregulated in the aged cells. Overall, it appears that not only the junction proteins themselves but also the GTPases that are involved in the junction assembly and regulation of junction proteins, such as N-cadherin, are diminished by 18 months in the rat. Due to these results we sought to determine whether or not the BTB was compromised at this age.

A previous study from our lab showed that the rat BTB was compromised by 24 months of age using a lanthium tracer [8]. In the present study we sought to determine at what stage the BTB becomes inefficient. Using a FITC-inulin tracer, we found that the BTB had already lost some of its integrity at 18 months, when spermatogenesis was still normal. The BTB gradually continued to lose its efficiency after 18 months; by 24 months it was unable to prevent the diffusion of substances into the lumen of the seminiferous tubules. It has been shown that the presence of spermatogonial stem cells alone is not sufficient for reinitiation of spermatogenesis [33] and that an intact BTB is required. Consequently, it is understandable that the testis cannot replace germ cells lost to, for example, DNA damage during aging, if the BTB is not intact. As we already saw a decrease in many of the junction-associated transcripts and proteins and a disruption of the BTB using immunofluorescenceat 18 months, we suggest that by this time point, the expression of GTPases and junction/cell adhesion proteins decreases, causing a gradual breakdown of the BTB that will allow potentially harmful substances to access germ cells. We propose that these, along with the accumulation of DNA damage we have previously reported [11], prevent the protection of the germ cells and cause the death and loss of germ cells resulting in testicular atrophy.
